# Supplementary material for: Distinctive neurophysiological correlates of sound onset and offset perception in humans
Source: J Physiol. 2026 Jan 31;604(21):8781–95. doi: 10.1113/JP289375 (PMC13725236; doi:10.1113/JP289375)
Supplement: Supplementary file 1 [file TJP-604-8781-s001.pdf]

The Journal of Physiology

Distinctive neurophysiological correlates of sound onset and offset perception in humans

Fatima Ali, Gabriela Bury, Adèle Simon, and Jennifer F. Linden
DOI: 10.1113/JP289375

Corresponding author(s): Jennifer Linden (j.linden@ucl.ac.uk)

The following individual(s) involved in review of this submission have agreed to reveal their identity: Anna Katharina Matkebauer (Referee #1)

Review Timeline:

Submission Date:	22-Jun-2025
Editorial Decision:	11-Aug-2025
Revision Received:	28-Nov-2025
Editorial Decision:	03-Dec-2025
Revision Received:	06-Dec-2025
Accepted:	09-Dec-2025

Senior Editor: Vaughan Macefield

Reviewing Editor: Conny Kopp-Scheinpflug

Transaction Report:

(Note: With the exception of the correction of typographical or spelling errors that could be a source of ambiguity, letters and reports are not edited. Depending on transfer agreements, referee reports obtained elsewhere may or may not be included in this compilation. Referee reports are anonymous unless the Referee chooses to sign their reports.)

Dear Dr Linden,

Re: JP-RP-2025-289375 "**Distinctive neurophysiological correlates of sound onset and offset perception in humans**" by Fatima Ali, Gabriela Bury, Adèle Simon, and Jennifer F. Linden

Thank you for submitting your manuscript to The Journal of Physiology. It has been assessed by a Reviewing Editor and by 2 expert referees and we are pleased to tell you that it is acceptable for publication following satisfactory revision.

The referee reports are copied at the end of this email.

Please address all the points raised and incorporate all requested revisions or explain in your Response to Referees why a change has not been made. We hope you will find the comments helpful and that you will be able to return your revised manuscript within 4 weeks. If you require longer than this, please contact journal staff: jp@physoc.org.

Your revised manuscript should be submitted online using the link in your Author Tasks Link Not Available. This link is accessible via your account as Corresponding Author; it is not available to your co-authors. If this presents a problem, please contact journal staff (jp@physoc.org). Image files from the previous version are retained on the system. Please ensure you replace or remove any files that are being revised.

If you do not wish to submit a revised version of your manuscript, you must inform our journal staff (jp@physoc.org) or reply to this email to request withdrawal. Please note that a manuscript must be formally withdrawn from the peer review process at one journal before it may be submitted to another journal.

TRANSPARENT PEER REVIEW POLICY: To improve the transparency of its peer review process The Journal of Physiology publishes online, as supporting information, the peer review history of all articles accepted for publication. Readers will have access to decision letters, including Editors' comments and referee reports, for each version of the manuscript, as well as any author responses to peer review comments. Referees can decide whether or not they wish to be named on the peer review history document.

ABSTRACT FIGURES: Authors are expected to use The Journal's premium BioRender account to create/redraw their Abstract Figures. Information on how to access this account is here: https://physoc.onlinelibrary.wiley.com/journal/14697793/biorender-access.

This will enable Authors to create and download high-resolution figures. If authors have used the free BioRender service, they can use the instructions provided in the link above to download a high-resolution version suitable for publication.

The link provided should only be used for the purposes of this submission. Authors will be charged for figures created on this account if they are not related to this manuscript submission.

LANGUAGE EDITING AND SUPPORT FOR PUBLICATION: If you would like help with English language editing, or other article preparation support, Wiley Editing Services offers expert help, including English Language Editing, as well as translation, manuscript formatting, and figure formatting at www.wileyauthors.com/eeo/preparation. You can also find resources for Preparing Your Article for general guidance about writing and preparing your manuscript at www.wileyauthors.com/eeo/prepresources.

RIGOUR AND REPRODUCIBILITY: We recommend authors consult The Journal's Rigour and Reproducibility requirements to ensure that their manuscript is suitable for publication: https://physoc.onlinelibrary.wiley.com/pb-assets/hub-assets/physoc/documents/TJP-Rigour-and-Reproducibility-Requirements.pdf

REVISION CHECKLIST:

Check that your Methods section conforms to journal policy: https://jp.msubmit.net/cgi-bin/main.plex?form_type=display_requirements#methods

Check that data presented conforms to the statistics policy: https://jp.msubmit.net/cgi-bin/main.plex?form_type=display_requirements#statistics

Upload a full Response to Referees file. To create your 'Response to Referees' copy all the reports, including any comments from the Senior and Reviewing Editors, into a Microsoft Word, or similar, file and respond to each point, using font or background colour to distinguish comments and responses and upload as the required file type.

Please upload two versions of your manuscript text: one with all relevant changes highlighted and one clean version with no changes tracked. The manuscript file should include all tables and figure legends, but each figure/graph should be uploaded as separate, high-resolution files. The journal is now integrated with Wiley's Image Checking service. For further details, see: https://www.wiley.com/en-us/network/publishing/research-publishing/trending-stories/upholding-image-integrity-wileys-image-screening-service

You may also upload:

- 'Potential Cover Art' for consideration as the issue's cover image
- Appropriate Supporting Information (Video, audio or data set: see https://jp.msubmit.net/cgi-bin/main.plex?form_type=display_requirements#supp).

We look forward to receiving your revised submission.

If you have any queries, please reply to this email and we will be pleased to advise.

Yours sincerely,

Vaughan Macefield
Senior Editor
The Journal of Physiology

REQUIRED ITEMS

- Author photo and profile. First or joint first authors are asked to provide a short biography (no more than 100 words for one author or 150 words in total for joint first authors) and a portrait photograph. These should be uploaded and clearly labelled together in a Word document with the revised version of the manuscript. See Information for Authors for further details.

- You must start the Methods section with a paragraph headed Ethical Approval. If experiments were conducted on humans, confirmation that informed consent was obtained, preferably in writing, that the studies conformed to the standards set by the latest revision of the Declaration of Helsinki and that the procedures were approved by a properly constituted ethics committee, which should be named, must be included in the article file. If the research study was registered (clause 35 of the Declaration of Helsinki), the registration database should be indicated, otherwise the lack of registration should be noted as an exception (e.g. The study conformed to the standards set by the Declaration of Helsinki, except for registration in a database). For further information see: https://physoc.onlinelibrary.wiley.com/hub/human-experiments.

- Please upload separate high-quality figure files via the submission form.

- Please ensure that any tables are editable and in Word format, and wherever possible, embedded in the article file itself.

- Please ensure that the Article File you upload is a Word file.

- Please include an Abstract Figure file, as well as the Figure Legend text within the main article file. The Abstract Figure is a piece of artwork designed to give readers an immediate understanding of the research and should summarise the main conclusions. If possible, the image should be easily 'readable' from left to right or top to bottom. It should show the physiological relevance of the manuscript so readers can assess the importance and content of its findings. Abstract Figures should not merely recapitulate other figures in the manuscript. Please try to keep the diagram as simple as possible and without superfluous information that may distract from the main conclusion(s). Abstract Figures must be provided by authors no later than the revised manuscript stage and should be uploaded as a separate file during online submission labelled as File Type 'Abstract Figure'. Please also ensure that you include the figure legend in the main article file. All Abstract Figures should be created using BioRender. Authors should use The Journal's premium BioRender account to export high-resolution images. Details on how to use and access the premium account are included as part of this email.

- Please include a full title page as part of your main article (Word) file, which should contain the following: title, authors, affiliations, corresponding author name and contact details, keywords, and running title.

EDITOR COMMENTS

Reviewing Editor:

Your manuscript has been reviewed by two experts in the field and both were enthusiastic about your work. There are are a few suggestions for you to tend to to further improve the manuscript. Perhaps you could also cross reference a very recent review in our Journal (Edeline and Liu) which has indicated the need and relevance of your type of study.

Please also see 'Required Items' above.

Senior Editor:

Thank you for submitting your manuscript to The Journal of Physiology, which has now been reviewed by two independent assessors and the Reviewing Editor. As you will see, both reviewers are enthusiastic about your contribution but present some minor suggestions for clarification.

I note that you already refer to the manuscript by Edeline and Liu submitted to the Journal.

Please state that SD is used when it first appears.

I look forward to receiving your revised manuscript in due course.

REFEREE COMMENTS

Referee #1:

The current EEG study investigates neural responses to sound on- and offsets in human listeners using two well established interval duration discrimination tasks. The authors carefully designed the tasks to overcome limitations of previous studies, by controlling the duration of white noise stimuli, the behavioural context (active vs. passive condition), the temporal predictability of stimuli, and by introducing cues for both on- and offset responses. Across the two EEG studies, the authors found that offset responses differ both in morphology and amplitude with offset responses being generally smaller than onset responses. Further, offset responses were dependent on the behavioural context (active vs. passive).

Overall, this is a very interesting and important paper examining neural responses to sound offsets. Sound offset responses are very understudied and often overlooked. The study has a relatively small sample size for EEG research, but all results are presented clearly, and the approach and analysis are transparent. I have a few general comments and remarks, some of which are a bit picky, but that simply reflects that the authors have already done a very good job. Some of the comments below are suggestions rather than essential revisions, and the authors should feel free to incorporate them as they see fit. Comments are listed roughly in order of appearance in the manuscript.

Major Comments:

Abstract: In general, the abstract is well-written. However, some parts are not easily understandable without reading the paper. For example:

- "Stimuli used in the active task were also presented under passive listening conditions" This sentence is not clear as the conditions have not been introduced yet. Something along the lines of "Tasks were designed to have both an active and passive component with identical stimuli" would be more suitable in the abstract.

- "...even though the roles of onset and offset cues as initial versus final markers of interval duration were reversed" A bit hard to grasp without reading the paper. Not sure it is needed in the abstract at all.

Methods:

- I am not sure it is required, but the paper does currently not have a typical participant section.

- The pre-processing steps are clearly described and generally very thorough. However, I did wonder whether any additional artefact rejection was applied at the epoch level. It is quite common in EEG research to exclude outlier trials based on criteria such as amplitude thresholds, kurtosis, or deviations beyond 2-3 SDs. If such a step was included, it would be helpful to provide some detail and indicate how many trials were retained on average per participant. If not, it might be worth clarifying whether all epochs were retained and, if so, whether any justification can be given for that approach.

Results:

- The authors have separate headings for each section, but for the first one. I think it would help the reader to have one for the first section as well, such as "Response magnitude for on- and offset responses". Further, the authors explain in the table that only for the P200 condition on- and offset responses could be reliably detected. I would suggest to add this information in the beginning of the results section as well. As it reads now, it sounds like it is a general phenomenon across all conditions and only when reading further it becomes clear this is not the case.

- Could the authors comment on whether the ERP waveforms were generally consistent across participants? Given the relatively small sample sizes in both studies, it would be helpful to know whether any individual outliers might have disproportionately influenced the grand average and/or results.

A more general comment: the abstract and introduction adopt a fairly confident tone regarding the importance of offset responses for speech perception. In contrast, the discussion is more cautious, referencing Colak et al. but generally more reserved. This is not necessarily something that needs changing, but I thought it worth flagging in case the authors wish to place greater emphasis in the discussion on the link to speech perception or their clinical importance.

Minor Comments:

Key summary: "Engagement in duration discrimination alters ERPs primarily for end-interval cues". Without reading the paper this key point is not entirely clear (e.g. what the authors mean by end-interval cues). If possible, this would benefit from re-writing or leaving out. The first three points in the key summary are very clear.

Introduction: "...Neurobiological studies in animals have recently elucidated distinct responses, neural mechanisms and pathways for central auditory processing..." When reading the first time it is not clear what the authors mean by "responses". I would suggest "...elucidated distinct neural responses, neural mechanisms and pathways...

p. 10, l 253: "Further investigation of onset and offset responses during active duration discrimination tasks in Study 1 and Study 2 revealed effects of behavioural context and cue order..." Could the authors clarify how this shows an effect of behavioural context? I thought the behavioural context was the comparison between active and passive condition, but here only the active condition is shown (so can only infer about the cue order).

Figures:

Figure 1: The figure is generally very clear. However, since the authors emphasise that tones and intervals were unpredictable and varied in duration, they might consider adjusting the figure to reflect that more visually. As it stands, it gives the impression that the sounds and intervals were identical across trials. While I see later in the text why the authors might have kept them similar, to allow comparison across Study 1 and 2, perhaps there is a way to reflect both the variability and the comparability in the figure.

Figure 2: The figure is generally very clear and clean. The ERP waveforms are on the same y-axis scale, which is helpful. However, the topographies use different scales. I would suggest using the same scale across all topoplots to make interpretation easier. It is already clear that sound offsets and N1 are smaller, so a uniform scale would not diminish the effect. Could the authors add in the plot description and/or in the text over what interval the topographies were averaged?

Figure 3/4/5: "The ERP difference waveforms (Figure 3, bottom row) confirmed

significant differences between Study 1 and Study 2..." The significant differences between the plots is not clear. It took me a bit to see the difference line in the top row. Rather than having it in the top plot, I would suggest to have it in the bottom row for the difference plot, on the same height and much more pronounced. It is also not in the description of figure 3, so I would add this. The same is true for Figure 4 & 5. The significant differences can hardly be seen.

Referee #2:

This manuscript describes a well-designed study with the goal to explore the evoked EEG responses of acoustical on- and off-sets. Sufficient time separation of the events allowed a clean dissection of the ERPs. The two decision tasks, where on-set and off-sets played different behavioral roles, illuminated clearly the differences in their EEG expression. This study significantly furthers the goal to identify the different roles that on- and -off-responses may play in perception, behavior and cognition, such as decision-making.

I have no main suggestions. One aspect, though, remains unclear to me - from a more general perspective since it is not explicitly part of the study. N100 and P200 are likely concerned with behavioral or cognitive bottom-up and top-down aspects, as stated in the Discussion. But both responses are also related to indicate perceptual changes that likely convey that information at a much faster time scale. One could imagine that interference with earlier periods of on- and off-responses (say <50ms) may impede the percept of the transition and content of two sequential stimuli. The continuity-illusion, or pulsation threshold paradigm, comes to mind. It could be hypothesized that forward- and simultaneous masking

of relevant aspects of the transition-responses is responsible for creating the illusion of a continues signals due to the absence of a fast transition response. How would that be reflected in the later transition responses studied here?

Minor points:

Figure 2: Indicate FZc and CMS locations on the maps

END OF COMMENTS

RESPONSE TO REFEREES

Reviewing Editor:

Your manuscript has been reviewed by two experts in the field and both were enthusiastic about your work. There are a few suggestions for you to tend to to further improve the manuscript. Perhaps you could also cross reference a very recent review in our Journal (Edeline and Liu) which has indicated the need and relevance of your type of study.

Please also see 'Required Items' above.

Senior Editor:

Thank you for submitting your manuscript to The Journal of Physiology, which has now been reviewed by two independent assessors and the Reviewing Editor. As you will see, both reviewers are enthusiastic about your contribution but present some minor suggestions for clarification.

I note that you already refer to the manuscript by Edeline and Liu submitted to the Journal.

Please state that SD is used when it first appears.

I look forward to receiving your revised manuscript in due course.

Many thanks to the editors and reviewers for their encouraging and very useful comments!

We have added an Abstract Figure and a new Appendix Figure 2 to show individual participant results and an Ethical Approval section at the start of the Methods section to align with journal policy. Additionally, we have strengthened the manuscript by adding a new Appendix Figure 1 showing that elevated post-P200 activity evoked by end-interval cues (onsets or offsets) in duration discrimination tasks is observed for both the first and second intervals, even though only the second interval is followed by a motor response. We have also made minor revisions to all the other figures in response to the reviewers' comments.

Regarding the request to refer to SD: we use confidence intervals rather than standard deviations in the paper, and this is explained at the end of the Methods section.

REFEREE COMMENTS

Referee #1:

The current EEG study investigates neural responses to sound on- and offsets in human listeners using two well established interval duration discrimination tasks. The authors

carefully designed the tasks to overcome limitations of previous studies, by controlling the duration of white noise stimuli, the behavioural context (active vs. passive condition), the temporal predictability of stimuli, and by introducing cues for both on- and offset responses. Across the two EEG studies, the authors found that offset responses differ both in morphology and amplitude with offset responses being generally smaller than onset responses. Further, offset responses were dependent on the behavioural context (active vs. passive).

Overall, this is a very interesting and important paper examining neural responses to sound offsets. Sound offset responses are very understudied and often overlooked. The study has a relatively small sample size for EEG research, but all results are presented clearly, and the approach and analysis are transparent. I have a few general comments and remarks, some of which are a bit picky, but that simply reflects that the authors have already done a very good job. Some of the comments below are suggestions rather than essential revisions, and the authors should feel free to incorporate them as they see fit. Comments are listed roughly in order of appearance in the manuscript.

Major Comments:

Abstract: In general, the abstract is well-written. However, some parts are not easily understandable without reading the paper. For example:

- "Stimuli used in the active task were also presented under passive listening conditions" This sentence is not clear as the conditions have not been introduced yet. Something along the lines of "Tasks were designed to have both an active and passive component with identical stimuli" would be more suitable in the abstract.

> Passive listening indicates no response was required of the participant; this was the only difference in the experiment setup between the conditions. We have now clarified this in the Abstract as follows: "Stimuli used in the active task were also presented under passive listening conditions with no participant response required."

- "...even though the roles of onset and offset cues as initial versus final markers of interval duration were reversed" A bit hard to grasp without reading the paper. Not sure it is needed in the abstract at all.

> To make our meaning more clear, we have changed this line to read, "...even though the functional roles of onsets and offsets as start-interval versus end-interval cues were reversed between the two tasks." We believe this point is important to include in the Abstract, to explain why we examined onset and offset responses in both silent interval in noise and noise interval in silence versions of the duration discrimination task.
>
> This comment made us realize that we had also been slightly inconsistent in our use of the term "cues" in the original manuscript. We have made minor corrections throughout the manuscript to refer more consistently to onset or

offset “events” (or “transients”) which can serve as start-interval or end-interval “cues”.

Methods:

- I am not sure it is required, but the paper does currently not have a typical participant section.

We have now added new subsections for Ethical Approval and Participants at the start of the Methods section.

- The pre-processing steps are clearly described and generally very thorough. However, I did wonder whether any additional artefact rejection was applied at the epoch level. It is quite common in EEG research to exclude outlier trials based on criteria such as amplitude thresholds, kurtosis, or deviations beyond 2-3 SDs. If such a step was included, it would be helpful to provide some detail and indicate how many trials were retained on average per participant. If not, it might be worth clarifying whether all epochs were retained and, if so, whether any justification can be given for that approach.

The statistical analysis used a Bayesian bootstrap estimation of 20% trimmed means with 95% highest density intervals (Rousselet et al., 2023; Wilcox & Rousselet, 2023). This non-parametric procedure accounts for artefacts that are typically excluded at the epoch level in more traditional statistical approaches to EEG data analysis. We have attempted to clarify this point by adding the following explanation to the Methods section: “This non-parametric Bayesian approach supersedes standard statistical approaches to EEG data analysis and operates on all recorded trials; it does not require prior exclusion of outlier trials based on pre-defined criteria such as fixed amplitude thresholds.”

Results:

- The authors have separate headings for each section, but for the first one. I think it would help the reader to have one for the first section as well, such as "Response magnitude for on- and offset responses". Further, the authors explain in the table that only for the P200 condition on- and offset responses could be reliably detected. I would suggest to add this information in the beginning of the results section as well. As it reads now, it sounds like it is a general phenomenon across all conditions and only when reading further it becomes clear this is not the case.

We have added the following subtitle for the initial paragraphs of the Results section: **P200 ERP component is a reliable measure for onset and offset responses**

- Could the authors comment on whether the ERP waveforms were generally consistent across participants? Given the relatively small sample sizes in both studies, it would be helpful to know whether any individual outliers might have disproportionately influenced the grand average and/or results.

Thank you for this important suggestion. We apologize for the omission of individual participant data from the original manuscript. We have added a new Appendix Figure 2 to show individual participant results for ERP waveforms recorded in active duration discrimination tasks in Study 1 and Study 2. We now use this figure both to show consistency of ERP waveforms across participants, and to illustrate that magnitude and morphology of average ERPs obtained using Matlab's standard EEGLAB function are similar to those we obtained using a non-parametric Bayesian estimation method (e.g., compare averages in Appendix Figure 2 to Appendix Figure 1).

A more general comment: the abstract and introduction adopt a fairly confident tone regarding the importance of offset responses for speech perception. In contrast, the discussion is more cautious, referencing Colak et al. but generally more reserved. This is not necessarily something that needs changing, but I thought it worth flagging in case the authors wish to place greater emphasis in the discussion on the link to speech perception or their clinical importance.

Thank you for this suggestion, which we have considered carefully. The main focus of this paper is examining the asymmetry of onset and offset responses in human EEG; this is relevant to ecologically valid theories of speech perception, but our data obviously do not include any speech perception measures. The cautious tone of our Discussion was motivated by the current lack of studies (aside from Colak et al.) directly relating offset responses with speech perception. We have revised the wording in the abstract and introduction to clarify that offset responses are thought to be important for speech perception, but that this hypothesis is underexplored. In particular, we have removed from the Introduction what was previously the first sentence of the second paragraph: "Brain-behaviour relationships in speech perception cannot be fully understood without consideration of the offset cue."

Minor Comments:

Key summary: "Engagement in duration discrimination alters ERPs primarily for end-interval cues". Without reading the paper this key point is not entirely clear (e.g. what the authors mean by end-interval cues). If possible, this would benefit from re-writing or leaving out. The first three points in the key summary are very clear.

The final key point has been rephrased to state: "Engagement in duration discrimination alters ERPs primarily at the end of intervals".

Introduction: "...Neurobiological studies in animals have recently elucidated distinct responses, neural mechanisms and pathways for central auditory processing..." When reading the first time it is not clear what the authors mean by "responses". I would suggest "...elucidated distinct neural responses, neural mechanisms and pathways...

We have rephrased this sentence to read:

“Neurobiological studies in animals have recently elucidated distinct neuronal and population responses, mechanisms and pathways for central auditory processing of sound offsets and onsets (Scholl et al., 2010; Anderson & Linden, 2016; Kopp-Scheinpflug et al., 2018).”

p. 10, l 253: "Further investigation of onset and offset responses during active duration discrimination tasks in Study 1 and Study 2 revealed effects of behavioural context and cue order..." Could the authors clarify how this shows an effect of behavioural context? I thought the behavioural context was the comparison between active and passive condition, but here only the active condition is shown (so can only infer about the cue order).

The behavioural context is whether the participant is listening out for gaps or noises (in two different backgrounds). To clarify, we have changed this in the text to read: “...revealed effects of behavioural context (i.e., participants listening out for gaps versus noises) and cue order (i.e., onsets or offsets appearing as start-interval versus end-interval cues)...”.

Figures:

Figure 1: The figure is generally very clear. However, since the authors emphasise that tones and intervals were unpredictable and varied in duration, they might consider adjusting the figure to reflect that more visually. As it stands, it gives the impression that the sounds and intervals were identical across trials. While I see later in the text why the authors might have kept them similar, to allow comparison across Study 1 and 2, perhaps there is a way to reflect both the variability and the comparability in the figure.

Thank you very much for this suggestion; we did not realize that the figure might be unintentionally misleading. The figure has now been modified to emphasise the length differences between the two intervals as well as the randomisation of durations across intervals and trials.

Figure 2: The figure is generally very clear and clean. The ERP waveforms are on the same y-axis scale, which is helpful. However, the topographies use different scales. I would suggest using the same scale across all topoplots to make interpretation easier. It is

already clear that sound offsets and N1 are smaller, so a uniform scale would not diminish the effect. Could the authors add in the plot description and/or in the text over what interval the topographies were averaged?

We have now clarified in the Figure 2 legend that these topographies are at the maximal peaks that have been recorded in Table 1, and the topoplots now all use the same scale.

Figure 3/4/5: "The ERP difference waveforms (Figure 3, bottom row) confirmed significant differences between Study 1 and Study 2..." The significant differences between the plots is not clear. It took me a bit to see the difference line in the top row. Rather than having it in the top plot, I would suggest to have it in the bottom row for the difference plot, on the same height and much more pronounced. It is also not in the description of figure 3, so I would add this. The same is true for Figure 4 & 5. The significant differences can hardly be seen.

Thank you for this very helpful suggestion for how to make the results more clear. Figures 3, 4 and 5 have now been edited with thicker significance bars on the difference plots in the bottom row, as suggested.

Referee #2:

This manuscript describes a well-designed study with the goal to explore the evoked EEG responses of acoustical on- and off-sets. Sufficient time separation of the events allowed a clean dissection of the ERPs. The two decision tasks, where on-set and off-sets played different behavioral roles, illuminated clearly the differences in their EEG expression. This study significantly furthers the goal to identify the different roles that on- and -off-responses may play in perception, behavior and cognition, such as decision-making.

I have no main suggestions. One aspect, though, remains unclear to me - from a more general perspective since it is not explicitly part of the study. N100 and P200 are likely concerned with behavioral or cognitive bottom-up and top-down aspects, as stated in the Discussion. But both responses are also related to indicate perceptual changes that likely convey that information at a much faster time scale. One could imagine that interference with earlier periods of on- and off-responses (say <50ms) may impede the percept of the transition and content of two sequential stimuli. The continuity-illusion, or pulsation threshold paradigm, comes to mind. It could be hypothesized that forward- and simultaneous masking of relevant aspects of the transition-responses is responsible for creating the illusion of a continues signals due to the absence of a fast transition response. How would that be reflected in the later transition responses studied here?

We agree that several auditory illusions can be related to onset and offset perception. It would be very interesting to study how masking of the offset transition, for example in a gap-detection paradigm with short silent gaps, might affect both early and late components of the transition response. However, in the present study, we intentionally tried to separate onset and offset responses cleanly in time using long gaps, so we cannot really address the reviewer's

question. In other ongoing work in the Linden lab, we are actively exploring this interesting question using very brief gaps in noise, so we hope to have more to say about this issue in the future..

Minor points:

Figure 2: Indicate FZc and CMS locations on the maps

FCz is now indicated on the topoplots in Figure 2, and the CMS location at the mastoids has been added in the Methods description of the setup.

Dear Dr Linden,

Re: JP-RP-2025-289375R1 "**Distinctive neurophysiological correlates of sound onset and offset perception in humans**" by Fatima Ali, Gabriela Bury, Adèle Simon, and Jennifer F. Linden

Thank you for submitting your manuscript to The Journal of Physiology. It has been assessed by a Reviewing Editor and we are pleased to tell you that it is acceptable for publication following satisfactory minor revision.

The review comments are copied at the end of this email.

Please address all the points raised and incorporate all requested revisions or explain in your Response to Referees why a change has not been made. We hope you will find the comments helpful and that you will be able to return your revised manuscript within 7 days. If you require longer than this, please contact journal staff: jp@physoc.org.

Your revised manuscript should be submitted online using the link in your Author Tasks Link Not Available. This link is accessible via your account as Corresponding Author; it is not available to your co-authors. If this presents a problem, please contact journal staff (jp@physoc.org). Image files from the previous version are retained on the system. Please ensure you replace or remove any files that are being revised.

If you do not wish to submit a revised version of your manuscript, you must inform our journal staff (jp@physoc.org) or reply to this email to request withdrawal. Please note that a manuscript must be formally withdrawn from the peer review process at one journal before it may be submitted to another journal.

TRANSPARENT PEER REVIEW POLICY: To improve the transparency of its peer review process The Journal of Physiology publishes online, as supporting information, the peer review history of all articles accepted for publication. Readers will have access to decision letters, including Editors' comments and referee reports, for each version of the manuscript, as well as any author responses to peer review comments. Referees can decide whether or not they wish to be named on the peer review history document.

ABSTRACT FIGURES: Authors are expected to use The Journal's premium BioRender account to create/redraw their Abstract Figures. Information on how to access this account is here: https://physoc.onlinelibrary.wiley.com/journal/14697793/biorender-access.

This will enable Authors to create and download high-resolution figures. If authors have used the free BioRender service, they can use the instructions provided in the link above to download a high-resolution version suitable for publication.

The link provided should only be used for the purposes of this submission. Authors will be charged for figures created on this account if they are not related to this manuscript submission.

LANGUAGE EDITING AND SUPPORT FOR PUBLICATION: If you would like help with English language editing, or other article preparation support, Wiley Editing Services offers expert help, including English Language Editing, as well as translation, manuscript formatting, and figure formatting at www.wileyauthors.com/eeo/preparation. You can also find resources for Preparing Your Article for general guidance about writing and preparing your manuscript at www.wileyauthors.com/eeo/prepresources.

RIGOUR AND REPRODUCIBILITY: We recommend authors consult The Journal's Rigour and Reproducibility requirements to ensure that their manuscript is suitable for publication: https://physoc.onlinelibrary.wiley.com/pb-assets/hub-assets/physoc/documents/TJP-Rigour-and-Reproducibility-Requirements.pdf

REVISION CHECKLIST:

Check that your Methods section conforms to journal policy: https://jp.msubmit.net/cgi-bin/main.plex?form_type=display_requirements#methods

Check that data presented conforms to the statistics policy: https://jp.msubmit.net/cgi-bin/main.plex?form_type=display_requirements#statistics

Upload a full Response to Referees file. To create your 'Response to Referees' copy all the reports, including any comments from the Senior and Reviewing Editors, into a Microsoft Word, or similar, file and respond to each point, using font or background colour to distinguish comments and responses and upload as the required file type.

Please upload two versions of your manuscript text: one with all relevant changes highlighted and one clean version with no changes tracked. The manuscript file should include all tables and figure legends, but each figure/graph should be uploaded as separate, high-resolution files. The journal is now integrated with Wiley's Image Checking service. For further details, see: https://www.wiley.com/en-us/network/publishing/research-publishing/trending-stories/upholding-image-integrity-wileys-image-screening-service

You may also upload:

- 'Potential Cover Art' for consideration as the issue's cover image
- Appropriate Supporting Information (Video, audio or data set: see https://jp.msubmit.net/cgi-bin/main.plex?form_type=display_requirements#supp).

We look forward to receiving your revised submission.

If you have any queries, please reply to this email and we will be pleased to advise.

Best wishes,

Vaughan Macefield
Senior Editor
The Journal of Physiology

REQUIRED ITEMS

- You must start the Methods section with a paragraph headed Ethical Approval. If experiments were conducted on humans, confirmation that informed consent was obtained, preferably in writing, that the studies conformed to the standards set by the latest revision of the Declaration of Helsinki and that the procedures were approved by a properly constituted ethics committee, which should be named, must be included in the article file. If the research study was registered (clause 35 of the Declaration of Helsinki), the registration database should be indicated, otherwise the lack of registration should be noted as an exception (e.g. The study conformed to the standards set by the Declaration of Helsinki, except for registration in a database). For further information see: https://physoc.onlinelibrary.wiley.com/hub/human-experiments.

EDITOR COMMENTS

Reviewing Editor:

The revised manuscript has further improved and is now a valuable contribution to the special issue on stimulus offsets. Please remember upon final corrections to add the accordance of your study with the declaration of Helsinki.

Senior Editor:

Thank you for submitting your revised manuscript, which will be accepted once you add reference to adhering to the Declaration of Helsinki in Methods.

END OF COMMENTS

RESPONSE TO REFEREES

Reviewing Editor:

Your manuscript has been reviewed by two experts in the field and both were enthusiastic about your work. There are a few suggestions for you to tend to to further improve the manuscript. Perhaps you could also cross reference a very recent review in our Journal (Edeline and Liu) which has indicated the need and relevance of your type of study.

Please also see 'Required Items' above.

[Additional comment added at final revision stage] Please remember upon final corrections to add the accordance of your study with the declaration of Helsinki.

Senior Editor:

Thank you for submitting your manuscript to The Journal of Physiology, which has now been reviewed by two independent assessors and the Reviewing Editor. As you will see, both reviewers are enthusiastic about your contribution but present some minor suggestions for clarification.

I note that you already refer to the manuscript by Edeline and Liu submitted to the Journal.

Please state that SD is used when it first appears.

I look forward to receiving your revised manuscript in due course.

Many thanks to the editors and reviewers for their encouraging and very useful comments!

We have added an Abstract Figure and a new Appendix Figure 2 to show individual participant results and an Ethical Approval section at the start of the Methods section to align with journal policy. Additionally, we have strengthened the manuscript by adding a new Appendix Figure 1 showing that elevated post-P200 activity evoked by end-interval cues (onsets or offsets) in duration discrimination tasks is observed for both the first and second intervals, even though only the second interval is followed by a motor response. We have also made minor revisions to all the other figures in response to the reviewers' comments.

Regarding the request to refer to SD: we use confidence intervals rather than standard deviations in the paper, and this is explained at the end of the Methods section.

We have also now added to the Ethical Approval section in Methods a reference to adherence with the Declaration of Helsinki. Apologies for this omission from previous revisions.

REFEREE COMMENTS

Referee #1:

The current EEG study investigates neural responses to sound on- and offsets in human listeners using two well established interval duration discrimination tasks. The authors carefully designed the tasks to overcome limitations of previous studies, by controlling the duration of white noise stimuli, the behavioural context (active vs. passive condition), the temporal predictability of stimuli, and by introducing cues for both on- and offset responses. Across the two EEG studies, the authors found that offset responses differ both in morphology and amplitude with offset responses being generally smaller than onset responses. Further, offset responses were dependent on the behavioural context (active vs. passive).

Overall, this is a very interesting and important paper examining neural responses to sound offsets. Sound offset responses are very understudied and often overlooked. The study has a relatively small sample size for EEG research, but all results are presented clearly, and the approach and analysis are transparent. I have a few general comments and remarks, some of which are a bit picky, but that simply reflects that the authors have already done a very good job. Some of the comments below are suggestions rather than essential revisions, and the authors should feel free to incorporate them as they see fit. Comments are listed roughly in order of appearance in the manuscript.

Major Comments:

Abstract: In general, the abstract is well-written. However, some parts are not easily understandable without reading the paper. For example:

- "Stimuli used in the active task were also presented under passive listening conditions" This sentence is not clear as the conditions have not been introduced yet. Something along the lines of "Tasks were designed to have both an active and passive component with identical stimuli" would be more suitable in the abstract.

> Passive listening indicates no response was required of the participant; this was the only difference in the experiment setup between the conditions. We have now clarified this in the Abstract as follows: “Stimuli used in the active task were also presented under passive listening conditions with no participant response required.”

- "...even though the roles of onset and offset cues as initial versus final markers of interval duration were reversed" A bit hard to grasp without reading the paper. Not sure it is needed in the abstract at all.

> To make our meaning more clear, we have changed this line to read, “...even though the functional roles of onsets and offsets as start-interval versus end-interval cues were reversed between the two tasks.” We believe this point is important to include in the Abstract, to explain why we examined onset and offset responses in both silent interval in noise and noise interval in silence versions of the duration discrimination task.
>
> This comment made us realize that we had also been slightly inconsistent in our use of the term “cues” in the original manuscript. We have made minor corrections throughout the manuscript to refer more consistently to onset or offset “events” (or “transients”) which can serve as start-interval or end-interval “cues”.

Methods:

- I am not sure it is required, but the paper does currently not have a typical participant section.

We have now added new subsections for Ethical Approval and Participants at the start of the Methods section.

- The pre-processing steps are clearly described and generally very thorough. However, I did wonder whether any additional artefact rejection was applied at the epoch level. It is quite common in EEG research to exclude outlier trials based on criteria such as amplitude thresholds, kurtosis, or deviations beyond 2-3 SDs. If such a step was included, it would be helpful to provide some detail and indicate how many trials were retained on average per participant. If not, it might be worth clarifying whether all epochs were retained and, if so, whether any justification can be given for that approach.

The statistical analysis used a Bayesian bootstrap estimation of 20% trimmed means with 95% highest density intervals (Rousselet et al., 2023; Wilcox & Rousselet, 2023). This non-parametric procedure accounts for artefacts that are typically excluded at the epoch level in more traditional statistical approaches to EEG data analysis. We have attempted to clarify this point by adding the following explanation to the Methods section: “This non-parametric Bayesian approach supersedes standard statistical approaches to EEG data analysis and operates on all recorded trials; it does not require prior exclusion of outlier trials based on pre-defined criteria such as fixed amplitude thresholds.”

Results:

- The authors have separate headings for each section, but for the first one. I think it would help the reader to have one for the first section as well, such as "Response magnitude for on- and offset responses". Further, the authors explain in the table that only for the P200 condition on- and offset responses could be reliably detected. I would suggest to add this information in the beginning of the results section as well. As it reads now, it sounds like it is a general phenomenon across all conditions and only when reading further it becomes clear this is not the case.

We have added the following subtitle for the initial paragraphs of the Results section:
P200 ERP component is a reliable measure for onset and offset responses

- Could the authors comment on whether the ERP waveforms were generally consistent across participants? Given the relatively small sample sizes in both studies, it would be helpful to know whether any individual outliers might have disproportionately influenced the grand average and/or results.

Thank you for this important suggestion. We apologize for the omission of individual participant data from the original manuscript. We have added a new Appendix Figure 2 to show individual participant results for ERP waveforms recorded in active duration discrimination tasks in Study 1 and Study 2. We now use this figure both to show consistency of ERP waveforms across participants, and to illustrate that magnitude and morphology of average ERPs obtained using Matlab’s standard

EEGLAB function are similar to those we obtained using a non-parametric Bayesian estimation method (e.g., compare averages in Appendix Figure 2 to Appendix Figure 1).

A more general comment: the abstract and introduction adopt a fairly confident tone regarding the importance of offset responses for speech perception. In contrast, the discussion is more cautious, referencing Colak et al. but generally more reserved. This is not necessarily something that needs changing, but I thought it worth flagging in case the authors wish to place greater emphasis in the discussion on the link to speech perception or their clinical importance.

Thank you for this suggestion, which we have considered carefully. The main focus of this paper is examining the asymmetry of onset and offset responses in human EEG; this is relevant to ecologically valid theories of speech perception, but our data obviously do not include any speech perception measures. The cautious tone of our Discussion was motivated by the current lack of studies (aside from Colak et al.) directly relating offset responses with speech perception. We have revised the wording in the abstract and introduction to clarify that offset responses are thought to be important for speech perception, but that this hypothesis is underexplored. In particular, we have removed from the Introduction what was previously the first sentence of the second paragraph: "Brain-behaviour relationships in speech perception cannot be fully understood without consideration of the offset cue."

Minor Comments:

Key summary: "Engagement in duration discrimination alters ERPs primarily for end-interval cues". Without reading the paper this key point is not entirely clear (e.g. what the authors mean by end-interval cues). If possible, this would benefit from re-writing or leaving out. The first three points in the key summary are very clear.

The final key point has been rephrased to state: "Engagement in duration discrimination alters ERPs primarily at the end of intervals".

Introduction: "...Neurobiological studies in animals have recently elucidated distinct responses, neural mechanisms and pathways for central auditory processing..." When reading the first time it is not clear what the authors mean by "responses". I would suggest "...elucidated distinct neural responses, neural mechanisms and pathways...

We have rephrased this sentence to read:

"Neurobiological studies in animals have recently elucidated distinct neuronal and population responses, mechanisms and pathways for central auditory processing of sound offsets and onsets (Scholl et al., 2010; Anderson & Linden, 2016; Kopp-Scheinpflug et al., 2018)."

p. 10, l 253: "Further investigation of onset and offset responses during active duration discrimination tasks in Study 1 and Study 2 revealed effects of behavioural context and cue

order..." Could the authors clarify how this shows an effect of behavioural context? I thought the behavioural context was the comparison between active and passive condition, but here only the active condition is shown (so can only infer about the cue order).

The behavioural context is whether the participant is listening out for gaps or noises (in two different backgrounds). To clarify, we have changed this in the text to read: "...revealed effects of behavioural context (i.e., participants listening out for gaps versus noises) and cue order (i.e., onsets or offsets appearing as start-interval versus end-interval cues)...".

Figures:

Figure 1: The figure is generally very clear. However, since the authors emphasise that tones and intervals were unpredictable and varied in duration, they might consider adjusting the figure to reflect that more visually. As it stands, it gives the impression that the sounds and intervals were identical across trials. While I see later in the text why the authors might have kept them similar, to allow comparison across Study 1 and 2, perhaps there is a way to reflect both the variability and the comparability in the figure.

Thank you very much for this suggestion; we did not realize that the figure might be unintentionally misleading. The figure has now been modified to emphasise the length differences between the two intervals as well as the randomisation of durations across intervals and trials.

Figure 2: The figure is generally very clear and clean. The ERP waveforms are on the same y-axis scale, which is helpful. However, the topographies use different scales. I would suggest using the same scale across all topoplots to make interpretation easier. It is already clear that sound offsets and N1 are smaller, so a uniform scale would not diminish the effect. Could the authors add in the plot description and/or in the text over what interval the topographies were averaged?

We have now clarified in the Figure 2 legend that these topographies are at the maximal peaks that have been recorded in Table 1, and the topoplots now all use the same scale.

Figure 3/4/5: "The ERP difference waveforms (Figure 3, bottom row) confirmed significant differences between Study 1 and Study 2..." The significant differences between the plots is not clear. It took me a bit to see the difference line in the top row. Rather than having it in the top plot, I would suggest to have it in the bottom row for the difference plot, on the same height and much more pronounced. It is also not in the description of figure 3, so I would add this. The same is true for Figure 4 & 5. The significant differences can hardly be seen.

Thank you for this very helpful suggestion for how to make the results more clear. Figures 3, 4 and 5 have now been edited with thicker significance bars on the difference plots in the bottom row, as suggested.

<u>Referee #2:</u>

This manuscript describes a well-designed study with the goal to explore the evoked EEG responses of acoustical on- and off-sets. Sufficient time separation of the events allowed a clean dissection of the ERPs. The two decision tasks, where on-set and off-sets played different behavioral roles, illuminated clearly the differences in their EEG expression. This study significantly furthers the goal to identify the different roles that on- and -off-responses may play in perception, behavior and cognition, such as decision-making.

I have no main suggestions. One aspect, though, remains unclear to me - from a more general perspective since it is not explicitly part of the study. N100 and P200 are likely concerned with behavioral or cognitive bottom-up and top-down aspects, as stated in the Discussion. But both responses are also related to indicate perceptual changes that likely convey that information at a much faster time scale. One could imagine that interference with earlier periods of on- and off-responses (say <50ms) may impede the percept of the transition and content of two sequential stimuli. The continuity-illusion, or pulsation threshold paradigm, comes to mind. It could be hypothesized that forward- and simultaneous masking of relevant aspects of the transition-responses is responsible for creating the illusion of a continues signals due to the absence of a fast transition response. How would that be reflected in the later transition responses studied here?

> We agree that several auditory illusions can be related to onset and offset perception. It would be very interesting to study how masking of the offset transition, for example in a gap-detection paradigm with short silent gaps, might affect both early and late components of the transition response. However, in the present study, we intentionally tried to separate onset and offset responses cleanly in time using long gaps, so we cannot really address the reviewer's question. In other ongoing work in the Linden lab, we are actively exploring this interesting question using very brief gaps in noise, so we hope to have more to say about this issue in the future..

Minor points:

Figure 2: Indicate FZc and CMS locations on the maps

> FCz is now indicated on the topoplots in Figure 2, and the CMS location at the mastoids has been added in the Methods description of the setup.

Dear Professor Linden,

Re: JP-RP-2025-289375R2 "**Distinctive neurophysiological correlates of sound onset and offset perception in humans**" by Fatima Ali, Gabriela Bury, Adèle Simon, and Jennifer F. Linden

We are pleased to tell you that your paper has been accepted for publication in The Journal of Physiology.

The last Word (or similar) version of the manuscript provided will be used by the Production Editor to prepare your proof. When this is ready you will receive an email containing a link to Wiley's Online Proofing System. The proof should be thoroughly checked and corrected as promptly as possible.

Authors should note that it is too late at this point to offer corrections prior to proofreading. Major corrections at proof stage, such as changes to figures, will be referred to the Editors for approval before they can be incorporated. Only minor changes, such as to style and consistency, should be made at proof stage. Changes that need to be made after proof stage will usually require a formal correction notice.

All queries at proof stage should be sent to: TJP@wiley.com.

Following copyediting and typesetting the accepted version will be published online.

Yours sincerely,

Vaughan Macefield
Senior Editor
The Journal of Physiology

IMPORTANT POINTS TO NOTE FOLLOWING ACCEPTANCE OF YOUR PAPER:

• IMPORTANT NOTICE ABOUT OPEN ACCESS: To assist authors whose funding agencies mandate immediate public access to published research findings, The Journal of Physiology allows authors to pay an Open Access (OA) fee to have their papers made freely available immediately on publication.

The Corresponding Author will receive an email from Wiley with details on how to register or log-in to Wiley Authors Services where you will be able to place an order.

• You can check if your funder or institution has a Wiley Open Access Account here: https://authorservices.wiley.com/author-resources/Journal-Authors/licensing-and-open-access/open-access/author-compliance-tool.html.

• TRANSPARENT PEER REVIEW POLICY: To improve the transparency of its peer review process, The Journal of Physiology publishes online as supporting information the peer review history of all articles accepted for publication. Readers will have access to decision letters, including Editors' comments and referee reports, for each version of the manuscript as well as any author responses to peer review comments. Referees can decide whether or not they wish to be named on the peer review history document.

• You can help your research get the attention it deserves! Check out Wiley's free Promotion Guide for best-practice recommendations for promoting your work at: www.wileyauthors.com/eeo/guide. You can learn more about Wiley Editing Services which offers professional video, design, and writing services to create shareable video abstracts, infographics, conference posters, lay summaries, and research news stories for your research at: www.wileyauthors.com/eeo/promotion.

• If you would like to receive our 'Research Roundup', a monthly newsletter highlighting the cutting-edge research published in The Physiological Society's family of journals (The Journal of Physiology, Experimental Physiology, Physiological Reports, The Journal of Nutritional Physiology and The Journal of Precision Medicine: Health and Disease), please click this link, fill in your name and email address and select 'Research Roundup':
https://www.physoc.org/journals-and-media/membernews

EDITOR COMMENTS

Thank you for attending to the Declaration of Helsinki statement.
